# Supplementary material for: Epigenetic remodeling by vitamin C potentiates plasma cell differentiation
Source: eLife. 2022 Sep 7;11:e73754. doi: 10.7554/eLife.73754 (PMC9451539; doi:10.7554/eLife.73754)
Supplement: Figure 7—source data 1. [file elife-73754-fig7-data1.zip › Chen et al- VC Fig.7 source data 1/Chen et al- VC Figure 7-source data-annotated.pdf]

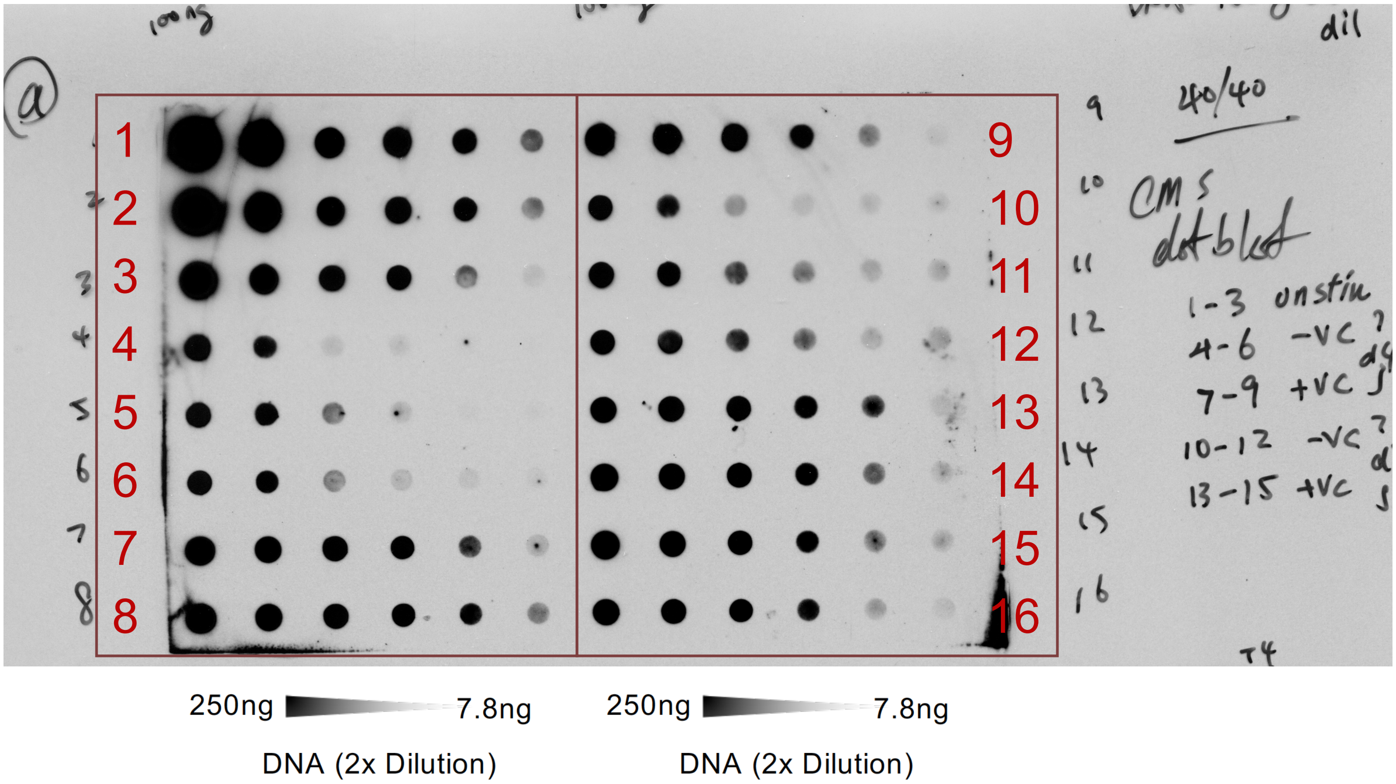


1. Naive rep 1
2. Naive rep 2
3. Naive rep 3
4. Day4$^{Mock}$ rep1
5. Day4$^{Mock}$ rep2
6. Day4$^{Mock}$ rep3
7. Day4$^{VC}$ rep1
8. Day4$^{VC}$ rep2
9. Day4$^{VC}$ rep3
10. Day7$^{Mock}$ rep1
11. Day7$^{Mock}$ rep2
12. Day7$^{Mock}$ rep3
13. Day7$^{VC}$ rep1
14. Day7$^{VC}$ rep2
15. Day7$^{VC}$ rep3
16. T4gt DNA (pos control)
