# Supplementary figures and images for: Epigenetic remodeling by vitamin C potentiates plasma cell differentiation
Source: eLife. 2022 Sep 7;11:e73754. doi: 10.7554/eLife.73754 (PMC9451539; doi:10.7554/eLife.73754)

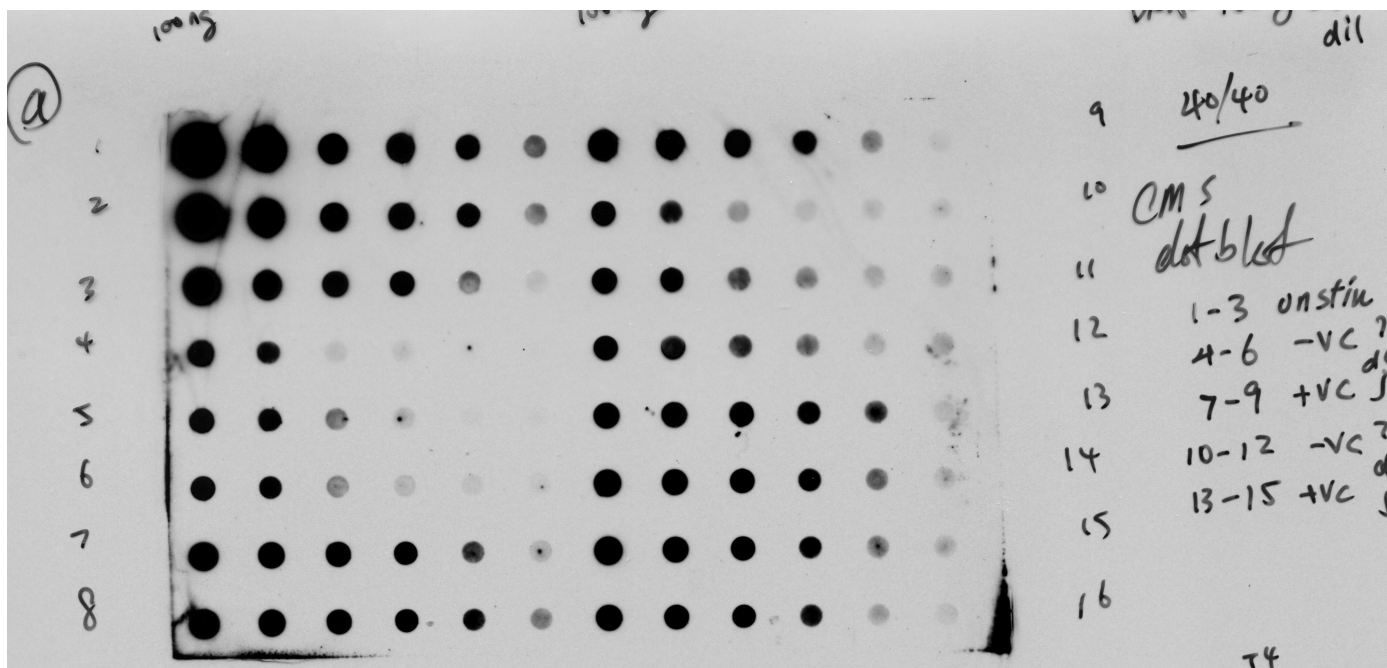

Supplement: Figure 7—source data 1. [file elife-73754-fig7-data1.zip › Chen et al- VC Fig.7 source data 1/Chen et al- VC Figure 7-source data 1.pdf]
